# Sleep disorders in functional neurological disorder - a systematic review and meta-analysis

**DOI:** 10.1007/s10072-024-07931-9

**Published:** 2024-12-30

**Authors:** Siddarth Kannan, Anirban Dutta, Abhijit Das

**Affiliations:** 1 University of Central Lancashire, Preston, UK; 2 Centre for Systems Modelling and Quantitative Biomedicine, University of Birmingham, Birmingham, UK; 3 Lancashire Teaching Hospitals NHS Foundation Trust, Preston, UK

**Keywords:** Functional neurological disorder, Sleep disorder, Sleep, Non-epileptic attack disorder (NEAD), WASO, ESS

## Abstract

**Introduction:**

Functional neurological disorders (FND) are conditions marked by disruptions in brain network function without structural abnormalities. Sleep disturbances, though under-researched, are commonly observed in FND patients and may worsen symptoms and overall health.

**Methods:**

This systematic review had been registered prospectively in PROSPERO with the registration number: CRD42023446306. Search of PubMed, MEDLINE, Embase, and Cochrane databases identified 218 articles. After removing duplicates and applying exclusion criteria, 9 studies were included in the final analysis.

**Results:**

The analysis showed a significant prevalence of sleep disorders among FND patients, with 58% reporting sleep disturbances, similar to other neuropsychiatric conditions. Studies on psychogenic non-epileptic seizures (PNES) indicated poorer subjective sleep quality and higher insomnia rates compared to epilepsy controls. Limited data on specific measures such as wake after sleep onset (WASO) and Epworth Sleepiness Scale (ESS) scores prevented definitive conclusions.

**Discussion:**

This review is the first systematic examination of sleep disorders in FND. The findings reveal a high prevalence of sleep disturbances, especially among PNES patients, correlating with lower quality of life and increased symptom severity. However, the heterogeneity of studies and limited reporting of specific sleep metrics weaken these conclusions. Further research is needed to investigate the direct impact of sleep quality on FND pathogenesis and management.

**Conclusion:**

Sleep disturbances are prevalent in FND patients and can significantly affect their quality of life. Increased awareness and routine evaluation of sleep in FND patients are recommended. Future studies should explore the relationship between sleep deprivation and FND to develop targeted therapeutic interventions.

**Supplementary Information:**

The online version contains supplementary material available at 10.1007/s10072-024-07931-9.

## Introduction

Functional neurological disorders (FND) are a spectrum of conditions that manifest primarily through disruptions in brain network function rather than structural changes and one of the most common causes of Neurology outpatient visits or in-patient admissions [1, 2]. Other chronic conditions like chronic pain and fatigue are also associated with FND and might be a core part of the spectrum [3]. These symptoms can be disabling, negatively affecting employment, activities of daily living, mood and quality of life, often to a greater extent than other neurological diseases [4, 5]. Understanding them is of crucial importance for developing a comprehensive therapeutic approach for management of FND which includes physiotherapy, occupational therapy and psychological approaches [6].

The presence of sleep disturbance has been noted in patients with FND, though limited research is currently present on the clinical relevance of sleep disturbance, its extent, broader characteristics, and impact [7]. Sleep disorders are known to be common in chronic diseases and can lead to worsening outcomes such as mood and quality of life [8, 9]. A study by Aasvik et al. (2018), found that the severity of insomnia had a substantial effect on working memory functioning in patients with comorbid Symptoms of Pain, Fatigue, and Mood Disorders [10]. Additionally, a recent study found that poor sleep (objective and subjective) predicted state dissociation and diminished state sense of agency, both of which are important in the pathomechanism of FND [9]. Unfortunately, sleep disorders are not often routinely evaluated for people with FND and the largest international online survey of FND people did not include it [10]. Hence it is important to identify whether sleep is a clinically relevant factor in pathogenesis of FND as therapeutic interventions targeting sleep disturbances may emerge as additional therapeutic avenues. It is important because various treatments such as cognitive behavioural therapy for insomnia (CBT-i), demonstrate effectiveness in enhancing sleep quality, subsequently influencing various outcomes including mood, quality of life, and severity of symptoms, across several neuropsychiatric and chronic disease cohorts [11, 12].

Therefore, the aim of our review is to analyse relevant literature reporting sleep disturbances/disorders in patients diagnosed with FND and if there is any significant correlation that could be addressed clinically in the management of FND.

## Methods

### Search strategy and inclusion criteria

**Fig. 1:**
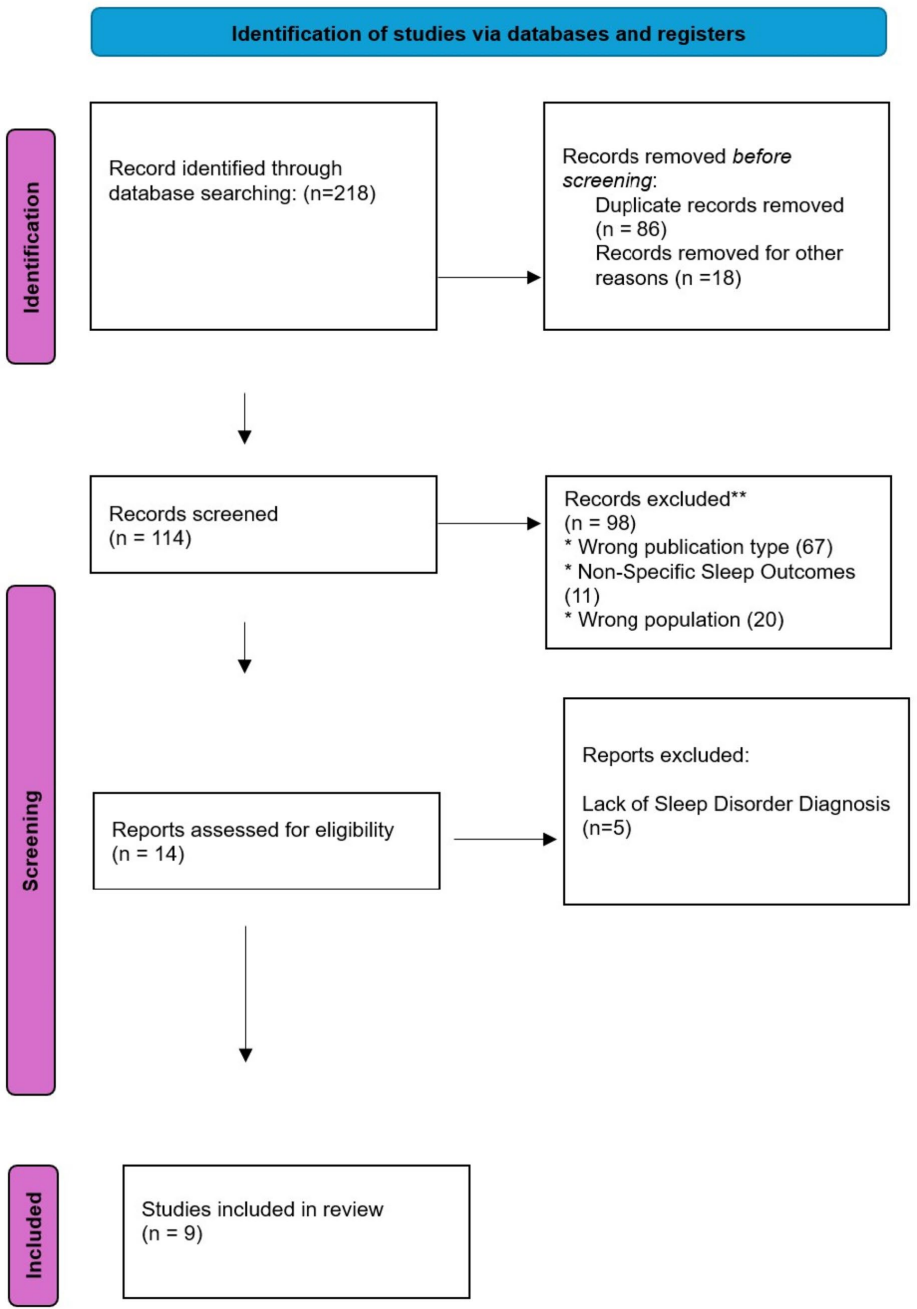
Preferred reporting items for systematic reviews and meta-analysis (PRISMA)

This systematic review was conducted in with the Preferred Reporting Items for Systematic Reviews and Meta-Analysis (PRISMA) guidelines as demonstrated in Fig. 1. This systematic review had been registered prospectively in the international register PROSPERO under the registration number: CRD42023446306.

We searched the PubMed, Embase, Cochrane and Medlinedatabases up to 31st December 2023 for eligible studies. The search strategy was based on the utilisation of MeSH (Medical Subject Headings) term and each keyword search of each database was conducted based on the Boolean operators AND and OR. The searched keywords utilised in the article screening process included the following: “Functional neurological disorder”, “Psychogenic non-epileptic seizures”, “Sleep disorders” “Functional motor disorder” (Supplementary Tables 1–3).

### Data extraction

The following data were extracted from each study: The first author’s last name, publication year, sample size, study design, type of FND, and methodology used to assess/monitor sleep. Data were extracted by one reviewer (SK) and checked for accuracy by a second reviewer (AD).

### Eligibility criteria

#### Inclusion criteria

Inclusion criteria were as follows: primary clinical studies involving adult patients who had a diagnosis of FND, systematic reviews looking at similar topic, available full text articles published in English, studies found on the Ovid MEDLINE, Cochrane Register of Clinical Trials, Embase and Pubmed databases.

#### Exclusion criteria

Non-original articles such as editorials, letters to the editor and conference abstracts were excluded, as well as articles looking at paediatric population and in languages other than English.

### Statistical analysis

Data was extracted via a Microsoft word document, then exported to R version 4.0 for analysis and figure generation. We calculated incidence of sleep disorders in patients with FND using the random-effects model which takes into account both within and between study variation (heterogeneity). Secondary analysis was performed comparing Wakefulness after sleep onset (WASO) and Epworth sleepiness scale (ESS) [13] in patients with FND compared to non-FND patients.

Heterogeneity between studies was evaluated using I^2^ statistics. Publication bias was assessed using Egger’s test and by inspection of funnel plots. Study quality was assessed using the Newcastle-Ottawa scale which ranks the studies on a scale from 0 to 9 based on the selection of the study population, comparability between cases and non-cases and the assessment of the outcome [14].

## Results

### Study selection

The database search yielded 218 articles (PubMed;110, MEDLINE; 76, Embase; 24 and Cochrane; 8). After the removal of 86 duplicates, 132 articles remained. These were assessed against a predetermined exclusion criteria by title and abstract. 18 were excluded due to inconsistent research objectives. Furthermore, the full text of the remaining 114 articles was attempted to be retrieved. The full text of 114 articles was assessed against the exclusion criteria majority of the papers were of wrong publication type including but not limited to literature reviews, case reports and abstracts; certain studies also reported non-specific sleep outcomes and wrong population. 9 articles were consistent with the inclusion criteria and so were included in this review.

### Baseline characteristics

The baseline characteristics of included studies are summarized in Table 1. Most common country of publication was the United Kingdom (4/9). Totally there were 1227 patients out of which 740 reported some form of sleep disorders.

**Table 1:** Baseline characteristics and outcomes measured for all the studies included in the final analysis

First author	Year	Number of patients with reported sleep disorders	Total number of patients diagnosed with FND	Type of FND	Study design
Nepožitek	2023	33	37	functional motor disorders (FMDs)	Comparative retrospective study
Sivathamboo	2019	51	93	psychogenic nonepileptic paroxysms & epileptic seizures	Comparative prospective study
Latreille	2019	13	17	psychogenic nonepileptic paroxysms & epileptic seizures	Comparative pilot prospective study
Bazil	2003	5	8	psychogenic nonepileptic paroxysms & epileptic seizures	Comparative prospective study
Graham	2017	152 + 18 (survey + clinical sample)	225 + 20 (survey + clinical sample)	FND	Cohort study
Tang	2016	97	149	Globus symptoms	Cohort study
Latreille	2018	114	149	psychogenic nonepileptic paroxysms & epileptic seizures	Comparative pilot prospective study
Popkirov	2019	11	22	psychogenic nonepileptic paroxysms & epileptic seizures	Comparative prospective study
Ducroizet	2023	246	527	FND	Cross-sectional study

### Sleep disorders

**Fig. 2:**
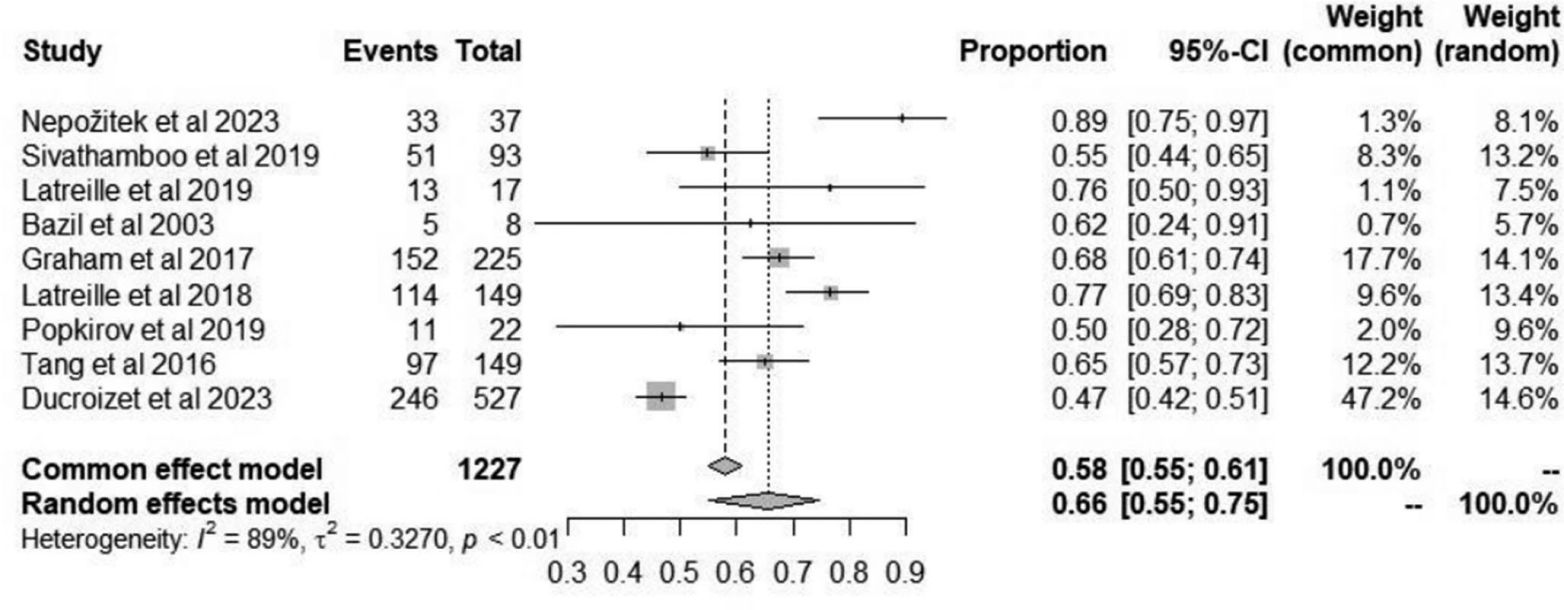
Forest plot depicting incidence of reported sleep disorders among patients FND

**Fig. 3:**
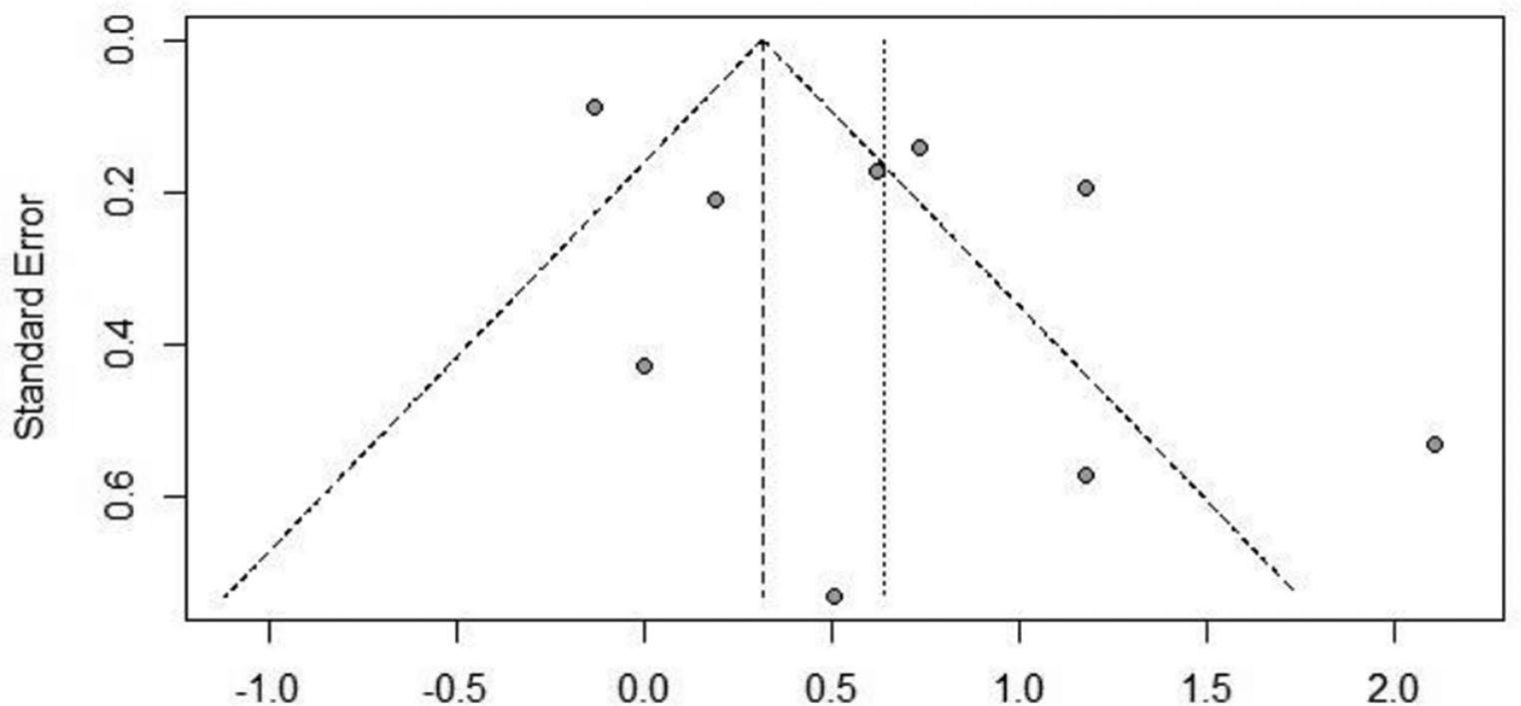
Funnel plot depicting outcome of Egger’s test

The overall proportion of patients with FND experiencing sleep disorders among all studies was 0.58 (95% CI: 0.55–0.61, I^2^ = 89%) (Fig. 2). The analysis did find that the presence of sleep disorders among patients with FND was significantly high (*p* < 0.01). There was no evidence of publication bias with Egger’s test, *p* = 0.08 (Fig. 3).

### Wakefulness after sleep onset (WASO)

**Fig. 4:**
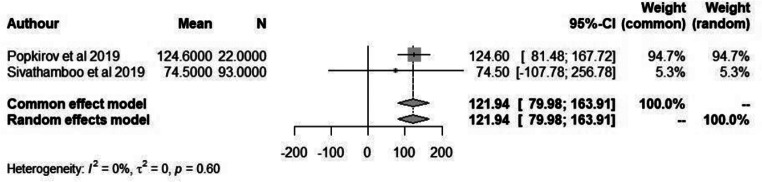
Forest plot depicting mean duration of WASO in patients with FND

**Fig. 5:**
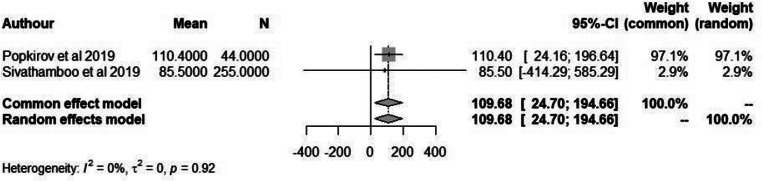
Forest plot depicting mean duration of WASO in non-FND patients

WASO time is an important parameter in measuring quality of sleep and a better reflection of sleep fragmentation. However, only 2 of the included studies reported WASO with an overall mean time of 121.9 min (95% CI: 80.0-163.9) in patients with FND compared to 109.7 (95% CI: 24.7- 194.7) minutes in non-FND patients (Figs. 4 and 5). However, due to the small sample size further studies would need to be conducted in order to determine its significance.

### Epworth sleepiness scale (ESS)

**Fig. 6:**
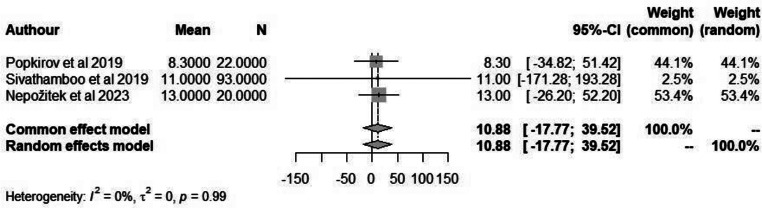
Forest plot depicting mean ESS score in patients with FND

**Fig. 7:**
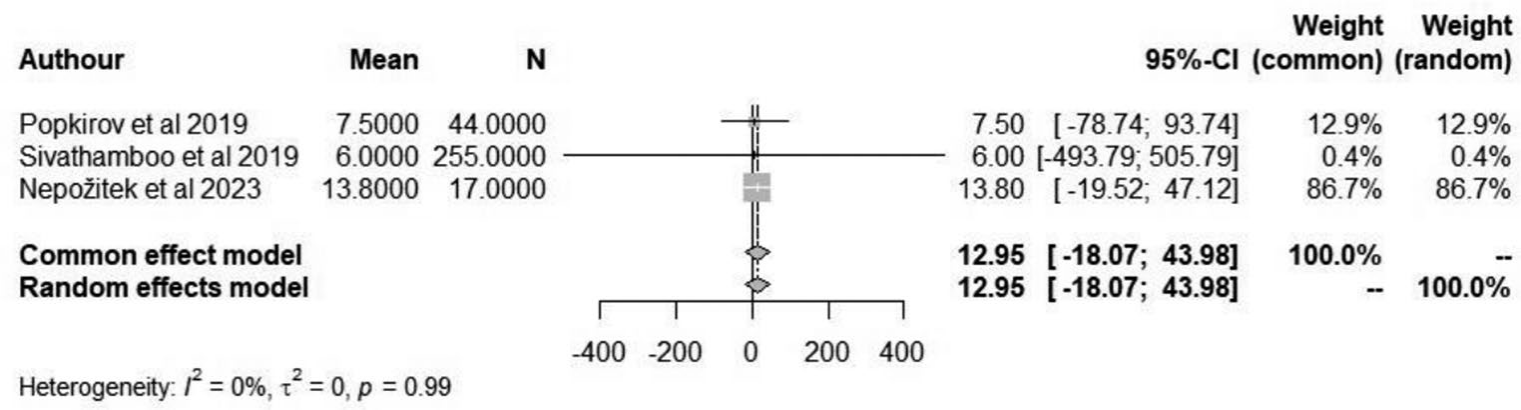
Forest plot depicting mean ESS score in non-FND patients

The ESS is a validated tool to assess the extent of subjective daytime sleepiness in eight different everyday life situations. The maximum score is 24, which indicates severe daytime sleepiness. This score suggests that a person is experiencing excessive daytime sleepiness, even in situations where they would typically be expected to stay alert. Despite the extensive use of the ESS in different types of patient populations and even in healthy persons, it has never been specifically validated in patients with FND. When applying a random-effects meta-analysis, we obtained a pooled mean of 10.88 (*P* = 0.99) in patients with FND compared to 12.95 in non-FND patients (Figs. 6 and 7).

### Risk of bias

**Fig. 8:**
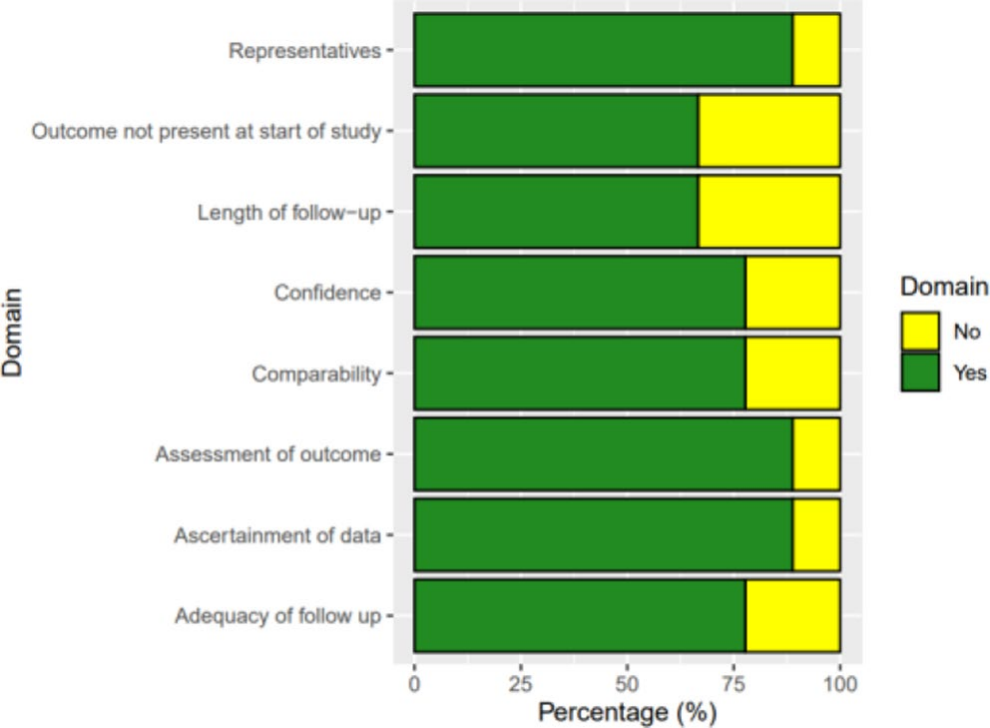
Bar graph depicting Risk of Bias among all included studies

The assessment of bias for retrospective cohort studies, using the Newcastle-Ottowa Scale, is detailed in Fig. 8. The mean score for all studies was 5.5 (out of a maximum total of 9), and no studies were classified to have a high risk of bias.

## Discussion

### Summary of findings

A meta-analysis was conducted on the included studies that reported sleep disturbances/disorders in patients with FND. We found that in patients with FND there were a significant proportion of individuals who experienced some form of sleep disorder. Further analysis was done to establish the possible link between sleep quality and FND by analysis studies that reported WASO and ESS score. However, due to the limited studies reporting this information no significant results were obtained.

### Comparison with literature

To our knowledge this is the first systematic review to address the presence of sleep disorders in patients with FND. Although there is a well-established relationship between sleep and psychiatric disorders [15, 16], there are very few data on sleep in FND. Some small studies (*n* < 20) have reported reduced subjective sleep quality as well as increased rapid-eye-movement sleep duration during polysomnography in patients with Psychogenic Non-epileptic seizures (PNES) [17, 18]. While the results slightly vary between studies; our study found that 58% of patients reported sleep disorders. This is similar to a recent study that found 46.7% of patients with FND experience sleep disturbances [19].

Of the 9 studies included 5 focused on patients with PNES. Latreille et al. (2018) compared sleep-wake patterns in a prospective observational study in PNES and epilepsy patients. Twenty-seven subjects were included in the study, of which 17 had PNES, and 10 were diagnosed epilepsy. Compared to epilepsy controls, the PNES patients showed increased latency of sleep onset (on average about 30 min longer than controls). Otherwise, both groups had a similar sleep architecture. However, the PNES patients subjectively had poorer sleep quality measured by Pittsburgh Sleep Quality Index (10.8 ± 5.1 versus 5.8 ± 2.9; *p* = 0.01; higher score indicates the worse quality of sleep) and met the clinical criteria for insomnia more often than patients with epilepsy (50% vs. 10%, *p* = 0.05) [20]. Compared to non-FND patients with FND were more likely to have mild to severe sleep changes, mostly shorter sleep length by up to 1–2 h, and difficulty falling asleep again. These changes in sleep patterns were linked with lower quality of life. These results propose that sleep disturbance is a more pronounced problem in PNES than in epilepsy [20, 21].

Popkirov et al. (2019) examined the occurrence of sleep disorders in a group of 22 PNES patients and 44 epilepsy patients. However, no significant differences were found between the two groups; in patients with epilepsy only three cases of mild or moderate obstructive sleep apnoea–hypopnea syndrome (OSAHS) (7%) and four cases of sleep-disordered breathing (SDB) (9%). Most of the patients were overweight or obese. In 22 patients with dissociative seizures, two (9%) had mild SDB, two (9%) had mild OSAHS and one (5%) had moderate OSAHS [22]. Similar results were obtained by Sivathamboo et al. (2019), Moderate to severe SDB was observed in 26.3% (67/255) of patients with epilepsy and 29.0% (27/93) of patient with PNES. Following adjustment for confounders, pathologic daytime sleepiness predicted moderate to severe SDB in epilepsy (odds ratio [OR] 10.35, 95% confidence interval [CI] 2.09–51.39; *p* = 0.004). In multivariable analysis, independent predictors for moderate to severe SDB in epilepsy were older age (OR 1.07, 95% CI 1.04–1.10; *p* < 0.001) and higher body mass index (OR 1.06, 95% CI 1.01–1.11; *p* = 0.029), and in PNES older age (OR 1.10, 95% CI 1.03–1.16; *p* = 0.002) [23]. Similar results were also obtained in patients with Functional Motor Disorder (FMD). Nepožitek et al. (2023) found that 23/37 (62%) of patients with FMD experienced sleep disturbances; 35% having restless legs syndrome; 49% obstructive sleep apnoea; and 8% periodic limb movements in sleep; however, the presence of these disorders was not correlated with subjective sleepiness. Patients with FMD with self-reported sleepiness reported higher fatigue (*p* = 0.002), depression (*p* = 0.002), and had longer sleep latencies in the MSLT (*p* < 0.001) compared to the patients with central hypersomnia [24]. Higher Wake After Sleep Onset (WASO) rates are a marker of sleep disruption and the Popkirov et al. paper highlighted this fact. WASO observed in patients with Functional seizures may not be coincidental. Research indicates a potential causal link between sleep disturbances and dissociative experiences [25]. Experimental studies have shown that acute sleep deprivation can increase dissociative symptoms in healthy individuals, as measured by self-report and cognitive tasks [26]. Insomniac patients also tend to score high on the Dissociative Experiences Scale, with these scores correlating with specific EEG findings [27]. The study by Bregman-Hai and Soffer-Dudek (2023) provides further support of this relationship between poor sleep and dissociation. The study found that poor sleep quality and posttraumatic symptoms (PTS) are independent pathways leading to dissociation and disruptions in the sense of agency. Specifically, individuals with low levels of PTS exhibited a stronger relationship between poor sleep (measured by WASO) and dissociative experiences, whereas this relationship was not observed in individuals with high levels of PTS. This suggests that sleep disturbances might contribute to dissociative experiences primarily in those with less severe posttraumatic symptoms via an independent pathway [28].

On the other hand, this opens up the possibility that targeting sleep disruption might be a therapeutic strategy to improve dissociative symptoms. Past research has shown that improving sleep quality in psychiatric patients has been associated with a reduction in dissociative symptoms [29]. However, we need a larger prospective study to explore this possibility in FND population.

### Limitations

Our study has several limitations. The presence of sleep disorders in the presence of FND was measured by the proportion of patients experiencing sleep disorders in each study. Due to this the heterogeneity (I^2^ = 89%) of studies included were high. In order to focus on the effect of sleep disturbances in FND a further analysis was done based on the ESS score and WASO time; however, due to only limited studies reporting this data no significant results were obtained. We also excluded full-text papers not available in English, restricting paper eligibility.

## Conclusion

Our study suggests that sleep disorder is common in people FND compared to other medical conditions. However, we need to ask more about sleep during our evaluation of people with FND. Due to limited studies reporting WASO we could not evaluate the direct relationship of sleep deprivation and FND but it remains a tantalizing prospect as a therapeutic target. Hence further research would need to be conducted to determine the significance of our results. Nevertheless, our study suggests that further awareness needs to be raised about sleep disturbances/disorders in patients with FND which could have significant impact on a quality of life of people with FND.

## Electronic supplementary material

Below is the link to the electronic supplementary material.

Supplementary Material 1

## Data Availability

All data used in this study is publicly available.
