# Supplementary material for: Sleep disorders in functional neurological disorder - a systematic review and meta-analysis
Source: Neurol Sci. 2024 Dec 30;46(4):1573–80. doi: 10.1007/s10072-024-07931-9 (PMC11920331; doi:10.1007/s10072-024-07931-9)
Supplement: Supplementary file 1 — Supplementary Material 1 [file 10072_2024_7931_MOESM1_ESM.docx]

**Supplementary Table 1.OVID Medline search strategy (Search performed 20^th^ October 2024).**

| **Search** | **Query** | **Results** |
| --- | --- | --- |
| 1 | exp Sleep disorder | 271125 |
| 2 | Functional neurological disorder.mp. | 902 |
| 3 | Psychogenicnon-epileptic seizures.mp. | 997 |
| 4 | Conversion disorder.mp. | 4217 |
| 5 | Functional Seizures.mp. | 291 |
| 6 | 2 OR 5 | 5476 |
| 7 | 1 AND 6 | 89 |
| 8 | Limit to Humans | 81 |
| 9 | Limit to English | 76 |

**Supplementary Table 2. OVID Embase search strategy (Search performed 20^th^ October 2024).**

| **Search** | **Query** | **Results** |
| --- | --- | --- |
| 1 | exp Sleep disorder | 221125 |
| 2 | Functional neurological disorder.mp. | 723 |
| 3 | Psychogenicnon-epileptic seizures.mp. | 778 |
| 4 | Conversion disorder.mp. | 1372 |
| 6 | Functional Seizures.mp. | 191 |
| 7 | 2 OR 6 | 2123 |
| 8 | 1 AND 7 | 39 |
| 9 | Limit to Humans | 31 |
| 10 | Limit to English | 24 |

**Supplementary Table 3. Pubmed search strategy (Search performed 20^th^ October 2024).**

| **Search** | **Query** | **Results** |
| --- | --- | --- |
| 1 | (“Functional neurological disorder” OR “FND” OR “Conversion disorder” OR "Nonepileptic Attack Disorder" OR "Psychogenic Nonepileptic Seizure " OR "Functional Seizures" OR "dissociative seizures" OR “NEAD” OR “PNES” OR “pseudoseizure” OR “Functional Motor Disorder”) AND  (“Sleep” OR “Sleep disorders” OR “Insomnia” OR “Somnolence” OR “Sleep Apnoea”) | 119 |
| 2 | Limit to English | 116 |
| 3 | Limit to Animals | 112 |
